# Chips and tags suggest plant-environment interactions differ for two alpine *Pachycladon* species

**DOI:** 10.1186/1471-2164-13-322

**Published:** 2012-07-19

**Authors:** Claudia Voelckel, Nicole Gruenheit, Patrick Biggs, Oliver Deusch, Peter Lockhart

**Affiliations:** 1 Institute of Molecular Biosciences, Massey University, Palmerston North, New Zealand; 2 Institute of Veterinary, Animal and Biomedical Sciences Massey University, Palmerston North, New Zealand

## Abstract

**Background:**

Expression profiling has been proposed as a means for screening non-model organisms in their natural environments to identify genes potentially important in adaptive diversification. Tag profiling using high throughput sequencing is a relatively low cost means of expression profiling with deep coverage. However the extent to which very short cDNA sequences can be effectively used in screening for candidate genes is unclear. Here we investigate this question using an evolutionarily distant as well as a closely related transcriptome for referencing tags. We do this by comparing differentially expressed genes and processes between two closely related allopolyploid species of *Pachycladon* which have distinct altitudinal preferences in the New Zealand Southern Alps. We validate biological inferences against earlier microarray analyses.

**Results:**

Statistical and gene annotation enrichment analyses of tag profiles identified more differentially expressed genes of potential adaptive significance than previous analyses of array-based expression profiles. These include genes involved in glucosinolate metabolism, flowering time, and response to cold, desiccation, fungi and oxidation. In addition, despite the short length of 20mer tags, we were able to infer patterns of homeologous gene expression for 700 genes in our reference library of 7,128 full-length *Pachycladon* ESTs. We also demonstrate that there is significant information loss when mapping tags to the non-conspecific reference transcriptome of *A. thaliana* as opposed to *P. fastigiatum* ESTs but also describe mapping strategies by which the larger collection of *A. thaliana* ESTs can be used as a reference.

**Conclusion:**

When coupled with a reference transcriptome generated using RNA-seq, tag sequencing offers a promising approach for screening natural populations and identifying candidate genes of potential adaptive significance. We identify computational issues important for the successful application of tag profiling in a non-model allopolyploid plant species.

## Background

Screening individuals that are naturally occurring across environmental and altitudinal gradients for differential gene expression is one approach proposed for the preliminary identification of candidate genes important in adaptive diversification and plastic responses [1-3]. Implementing this approach may involve large numbers of comparisons and thus requires a cost effective means of expression profiling.

Two variations on high throughput sequencing of short cDNA fragments – RNA-seq and tag profiling – both require small amounts of RNA, and have the potential to identify low abundance transcripts and/or provide for analysis of a large number of samples [4]. Unlike microarrays, there are no background and cross-hybridisation problems and there is the potential to interrogate any transcript that is expressed as opposed to the interrogation of pre-selected probes [5]. These approaches are potentially available for any organism.

Studies have already demonstrated that sequencing tags (typically 20–36 bases of cDNA) produces more robust results and detects more differentially expressed genes than several different microarray platforms, particularly when using a con-specific reference genome/transcriptome to which tags can be aligned [5]. For example, in one tag profiling study with mice that used a con-specific reference, the collective percentage of ambiguously or non-mapping and thus non-informative tags was as little as 12% [5]. However, even with rapidly increasing sequencing capacity, decreasing sequencing costs, and initiatives such as the 1kp project (http://www.onekp.com/) most non-crop and non-model species still lack phylogenetically close reference transcriptomes/genomes. An important question is whether or not a more distantly related transcriptome can be used effectively when profiling short RNA/cDNA sequences.

Sequence tags (20–36 bp) also pose analytical challenges [5,6] and while tag profiling protocols have been developed on several new generation sequencing platforms [7,8], their principles of analysis differ. Here we show and discuss the complex nature of tag sequences generated using the IIlumina Digital Gene Expression (DGE) – tag profiling protocol [9]. We profile natural populations of two closely related species – *Pachycladon fastigiatum* and *Pachycladon enysii* - which are members of a small allopolyploid genus (2n = 20), native to the Southern Alps of New Zealand. All *Pachycladon* species formed very recently (< 1 mya) [10] and presumably this has been an adaptive radiation [11]. We use expression profiling as a means to predict differences in adaptive traits between *Pachycladon* species. *P. fastigiatum* and *P. enysii* are known to differ in their altitudinal preferences and in their glucosinolate metabolism [11,12]. Differences in glucosinolate biosynthesis and hydrolysis had been predicted by a heterologous microarray study and subsequently confirmed by HPLC. In this tag profiling study, we analyse the same cDNA samples that were previously investigated with *Arabidopsis* 70mer oligonucleotide microarrays [12].

We evaluate how effective 20mer tag sequencing is for identifying candidate genes and biological processes when (a) a distant but well annotated transcriptome (TAIR10 release of *Arabidopsis thaliana*) is used as a reference, (b) when a reference transcriptome for *P. fastigiatum* generated with RNA-seq is used, and (c) when partial sequences instead of full length transcripts are used.

## Methods

### Sample preparation

RNA from three native populations of *P. enysii* (New Zealand Southern Alps: Mount Potts, Mount Hutt, Broken River) and *P. fastigiatum* (New Zealand Southern Alps: Ohau Ski field, Mount Hodgkinson, Twin Stream) was isolated as described in [12]. RNAs from multiple accessions of each species were pooled [12] and underwent sample preparation according to manufacturer’s instructions (DGE-Tag Profiling *Dpn*II Sample Prep Kit, Illumina Inc., San Diego, USA). mRNA was isolated from total RNA and *Dpn*II-restricted to generate *Dpn*II-anchored tags which were then enriched for sequencing. After tag library construction, libraries were titrated resulting in three flow cell lanes being loaded for each species. Cluster generation and sequencing were conducted according to Illumina protocols (DGE-Tag Profiling *Dpn*II Cluster Generation Kit, 18 Cycle Solexa Sequencing Kit, Illumina Inc., San Diego, USA). The sequence reads are available at the ArrayExpress database (http://www.ebi.ac.uk/arrayexpress) under the accession number E-MTAB-610.

### Reference genes

Four sets of reference genes were used for mapping. First, 6,428 full length reference genes obtained by Illumina short read sequencing of *P. fastigiatum* were extracted from an EST library [13]. Two homeologous copies were found for 700 of these genes resulting in a total set of 7,128 *P. fastigiatum* reference ESTs (Additional file 1). Their *A. thaliana* homologues were identified using BLAST [14] and extracted from the TAIR10 database [15] and represent the second set of reference genes. The third set of reference genes contained all contigs longer than 200 bp in the *P. fastigiatum* EST library (9,636,919, [13]), while a fourth set consisted of the cDNAs of all 33,602 gene models in the TAIR10 database.

### Read quality, mapping and counting

The base calling quality for each position in 18 bp reads from all six lanes was assessed using the program DynamicTrim [16]. Since the sequencing protocol artificially added two nucleotides to the end of each read, these two bases were clipped giving high quality tags of 16 bp in length (DGE-Tag Profiling *Dpn*II Sample Prep Kit, Illumina Inc., San Diego, USA). As all tags must begin with a *Dpn*II restriction site that cleaves 3′ of GATC, the sequence GATC was added to the beginning of each read resulting in a length of 20 bases for every tag. These tags were mapped for each individual lane to the full length ESTs (7,128) of *P. fastigiatum* without allowing any mismatches (P0) as well as allowing for one mismatch when mapping the tags of *P. enysii* (P1), and to the corresponding *A. thaliana* TAIR10 orthologues allowing no (A0), one (A1) and two (A2) mismatches using Bowtie v. 0.12.5 [17]. The tags were also mapped without (PL0) and with one mismatch in the *P. enysii* tags (PL1) to all available contigs of *P. fastigiatum* (9,636,919) as well as to all cDNA sequences of the TAIR10 database (33,602) allowing for no (AL0), one (AL1) and two (AL2) mismatches. All reads that mapped to more than one gene locus were discarded whereas reads mapping to both homeologous copies were counted once. When reads were mapped against all *P. fastigiatum* contigs, a read was counted if it uniquely mapped to a contig that was homologous to a specific *Arabidopsis* gene. If several contigs representing the same gene had reads mapping to them, the read counts were added to obtain the total count for the gene.

An in silico *Dpn*II digestion of the 7,128 *P. fastigiatum – A. thaliana* orthologues was carried out to reveal the distribution of *Dpn*II sites in reference genes. This distribution is shown in Additional file 2 and indicates that *Dpn*II sites were absent in some genes and occurred more than 20x in 66 *P. fastigiatum* and 50 *A. thaliana* genes.

According to the Illumina *Dpn*II sample preparation protocol, only the tag anchored to the 3′ most *Dpn*II site should remain attached to the bead and be sequenced [9]. However, for most reference genes, tags mapping to several *Dpn*II sites per gene were recovered with the 3′ most tag often not being the most abundant tag (data not shown). This phenomenon has been previously observed and ascribed to both incomplete digestion by *Dpn*II as well as the presence of multiple polyadenylation sites per gene [18]. Therefore, when obtaining counts for individual gene loci, instead of counting only the 3′ most tag or the most abundant tag, we summed all tags that mapped to a locus regardless of their positions within the gene. This also compensated for the loss of tag positions due to sequence divergence when using heterospecific reference transcriptomes of *A. thaliana*.

In cases where tags mapped to the same position, but one group of tags was oriented in the forward direction and one group in the reverse direction, both positions and counts were combined into one. Our goals were to 1) quantify the expression of a particular gene locus irrespective of alternative splicing variants or homoelogous copies making up that locus, 2) compare the expression of genes between this tag profiling and a previous microarray study and 3) quantify the expression of separate homeologous gene copies present at the same locus. Thus, we first distinguished between locus-specific tags (tags mapping to both copies of a gene locus) and locus copy-specific tags (tags mapping uniquely to one of two homeologous copies). Then, for goals 1 and 2, locus-specific and locus copy-specific tags of both copies were added to obtain the locus count. For the analysis of homeologous copies (goal 3) see paragraph below.

### Analysis of differentially expressed genes (DEGs)

Differential gene expression analyses were made with R using the Bioconductor software package edgeR [19]. The methods implemented in edgeR [20] assume tag count data to be described by an overdispersed negative binomial distribution. A maximum likelihood procedure was used to estimate common dispersion conditional on total tag counts (see Additional file 3 for respective library sizes); log_2_(*propE*) and log_2_(*propF*) represent corrected tag proportions for *P. enysii* and *P. fastigiatum*, respectively; *propE* and *propF* reflect count averages across the three replicate lanes per species. An exact test analogous to Fisher’s exact test, but for overdispersed data, was used to assess differential gene expression. Conditioning on the pseudo-data totals over all libraries, the test calculates the probability of observing sample totals as or more extreme than that observed in the experiment for each gene (*p*-value). P-values were adjusted for multiple testing using the Benjamini-Hochberg procedure. Criteria for differential expression were an absolute *M* (log_2_(*propE*) - log_2_(*propF*)) value > 0.58 (= fold change of 1.5) and an adjusted *p*-value < 0.05. This criterion was applied to make interpretation of results comparable with those of a previous microarray study that used a similar threshold. A total of 10 datasets were analysed. Four datasets resulted from mapping the tags against the collection of full length and partial *P. fastigiatum* ESTs either allowing for no mismatch or one mismatch with the *P. enysii* tags. Six datasets resulted from mapping tags against *A. thaliana* ESTs orthologous to the full length *P. fastigiatum* ESTs and against the complete TAIR10 database allowing for zero, one or two mismatches.

### Analysis of agreements and discrepancies between sets of DEGs

**Figure 1:**
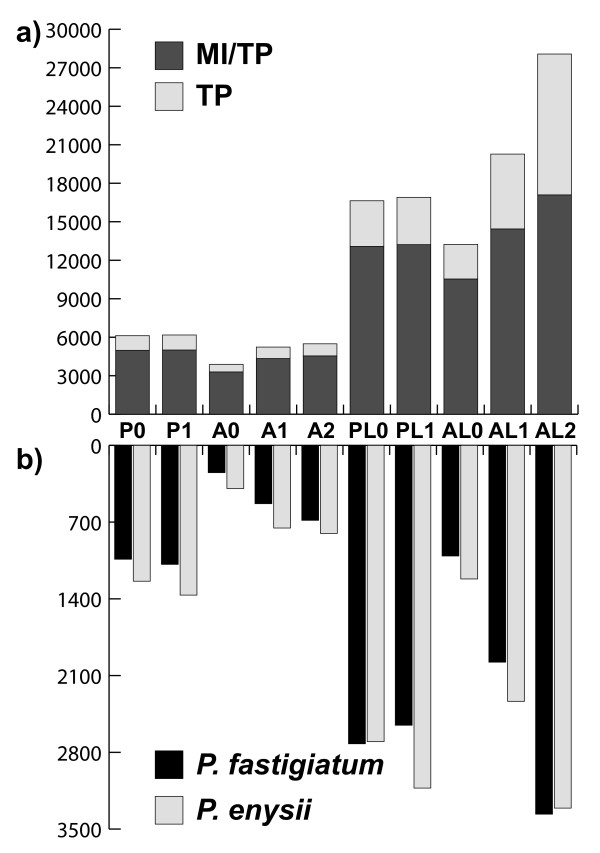
**a) Heatmap showing percentages of overlap in numbers of DEGs between different analyses using different sets of reference genes as well as between tag profiling and microarray analyses.** Percentages between different reference sets ranged from 44% to 90% indicating that the choice of the reference set greatly affected what genes were being identified as differentially expressed. Percentages of overlap between microarray analysis and tag profiling were higher with the *P. fastigiatum* reference sets than with the *A. thaliana* reference sets in both species (49-60% vs. 21-46%) This indicates that mapping tags to *P. fastigiatum* is preferable to mapping tags to *A. thaliana*. All overlaps between microarray and tag profiling analyses were statistically significant (denoted by stars). **b**) **Heatmap showing percentages of contradictory results obtained using different sets of reference genes and between tag profiling and microarrays (= percentage of genes up-regulated in *P. enysii* with one method and *P. fastigiatum* with another method).** Disagreements between different tag profiling datasets ranged from 0% to 11% indicating the detection of false positives with almost all approaches. All disagreements between tag profiling and microarray analyses were not statistically significant. Also, disagreements did not differ between *P. fastigiatum* and *A. thaliana* reference sets (6-12% vs 0-14%). For abbreviations of different sets of reference genes see Aditional file 3 and for calculation of percentages and statistical significance see methods.

To determine to which degree similar DEGs are identified between the ten different tag profiling datasets as well as tag profiling and a previous microarray analysis we intersected lists of DEGs for all treatments shown in Figure 1. First, we subtracted from the number of DEGs of the first treatment the number of genes not surveyed by the second treatment. For example, 1,034 of 1,238 genes up-regulated in *P. enysii* with tag profiling (P0) were also surveyed by microarrays while the remaining 234 were not. Similarly, 110 of the 305 genes up-regulated in *P. enysii* with microarrays were also surveyed by tag profiling (P0) while the remaining 195 were not. Hence, the overlap was calculated between the corrected DEG values, namely 1,034 and 110 genes and equalled 56 genes. This means that 51% of the microarray results (56 of 110 genes) were confirmed by tag profiling (P0). We always divided the number of overlapping genes by the smaller of the two corrected number of DEGs. This allowed for a straightforward comparison of percentages (Figure 1a).

In addition to cases where two different datasets identified similar DEGs we also investigated cases for which two methods contradicted one another, i.e. cases for which the first method identifies a gene as up-regulated in *P. enysii* whereas the second method identifies the same gene as up-regulated in *P. fastigiatum* and vice versa. To calculate disagreements we intersected ‘opposite’ lists. First, we subtracted from the number of DEGs of one method the number of genes not surveyed by the other method. For example, the number of genes up-regulated in *P. enysii* with microarrays and in *P. fastigiatum* with tag profiling was 305 and 1,038, respectively. However, only 110 and 844 of those were surveyed by the other analysis. Hence an overlap between the latter of 6 genes means that 5.5% (6 of 110) of the microarray results were contradicted by tag profiling (Figure 1b).

### Comparison with microarrays

We applied a statistical test to evaluate agreements and disagreements in the results obtained for differential expression from our microarray and tag profiling analyses. Using a resampling approach, we calculated a null frequency distribution to determine how likely it was to observe similar and different patterns of gene expression between platforms by chance. *Y* was the number of genes surveyed for differential expression by both platforms (the exact value of *Y* differed in separate analyses dependent on which reference transcriptome and mapping strategy was used for tag profiling). From *Y*, we jackknife resampled *n* elements (the number of genes found to be differentially expressed in the tag profiling analyses) and *m* elements (the number of genes found to be differentially expressed in the microarray analyses). We recorded the number of elements that were common to (and also different between) both resampled datasets. This sampling process was repeated a total of 10,000 times for each analysis so that an appropriate null frequency distribution could be generated. The actual number (*z*) of up-regulated and down-regulated genes suggesting concordance or disagreement between the tag profiling and microarray results were then compared against values of the null frequency distribution to determine significance. The test was performed using a MySQL database and Perl.

### Gene-annotation enrichment analysis

The loci found to be up-regulated in *P. enysii* and *P. fastigiatum* as well as loci with one homeologous copy up-regulated in *P. enysii* and the other in *P. fastigiatum* were subjected to a gene-annotation enrichment analysis using agriGO [21]. 6,122 reference genes that contained a *Dpn*II restriction site were used as population background for the smaller datasets, while all available TAIR10 cDNA sequences were used as population background for the large datasets. AgriGO analyses were performed for all ten datasets. In addition, a DAVID analysis [22,23] was done for dataset P0. Gene annotations were compared to the curated GO database for biological processes (GOTERM_BP_FAT) and the KEGG database and the classification stringency was set to medium. DAVID analyses identify clusters of GO terms that are enriched for either species as well as enriched GO terms. For each GO term, a Fisher’s exact test was performed to determine if a GO term occurs significantly more often in the respective set of up-regulated genes than in the EST library used as population background. For each cluster of GO terms, an enrichment score was determined by calculating the minus log transformation of the geometric mean of all *p*-values for the GO terms in that cluster. A score greater than or equal to 1.3 is equivalent to 0.05 on a non-log scale and considered significant. Significant clusters and GO terms for each species are summarized in Additional file 4.

### Analysis of differential expression between homeologous copies

We examined differential gene expression for 700 full length homeologous gene pairs in the *P. fastigiatum* library. Of these, all loci for which the number of locus copy-specific tags was greater than five and exceeded the number of copy-unspecific tags by at least fivefold were analysed. 379 loci (758 sequences) met this criterion. For five of the 700 loci, three divergent sequences were found in the EST library (15 sequences total). In all cases homeologous copies were less than 95% similar, while a further putative paralogue was less than 90% similar*.* This interpretation of paralogy is consistent with the presence of a duplicated gene in the genome of *Arabidopsis lyrata* (AT1G54030). The counts for these five additional sequences were added to this dataset now comprising 773 sequences (758 + 15) which could be analysed for copy-specific differential expression.

## Results

### Read quality assessment

We refer to the processed reads of each lane as ‘tags’, all distinct tags as ‘unique tags’ and the number of occurrences of each unique tag as ‘tag counts’. Tags that map to only one locus are called unambiguous tags even if they map to both copies of one locus.

For four of the six lanes on our flow cell more than 90% of the reads met a high quality threshold; only in the lanes with the highest concentrations (PE3 and PF3) was a significant number of reads (~20%) discarded (Additional file 3). Thus the overall quality of the data was very high. In the following we report the results for lanes PE1, PE2 and PE3; the results for lanes PF1, PF2, and PF3 were similar (data not shown). We found 191,776, 276,919 and 278,657 unique 20 bp tags (16 bp tag plus GATC restriction site) in PE1, PE2, and PE3, respectively. 58,580 (30.6%) of the unique tags found in PE1 were not found in PE2 and PE3, and 116,547 (42.1%) and 117,740 (42.3%) of the unique tags were only present in PE2 and PE3, respectively. There were 96,426 unique tags common to all three PE lanes.

### Tag mapping to *P. fastigiatum* ESTs

**Figure 2:**
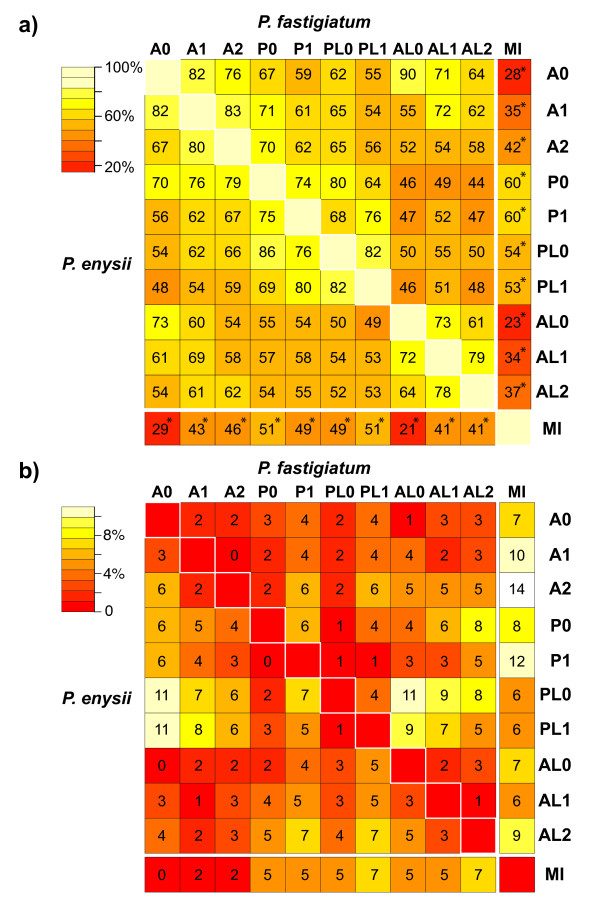
**a) Number of genes investigated with different sets of reference genes. Bar heights indicate the number of genes which were surveyed with tag profiling (TP, light shading) and a previous microarray study (MI, dark shading).** With the small reference sets, increasing the number of mismatches in mapping *Pachycladon* tags to *Arabidopsis* TAIR10 protein coding sequences increased the number of investigated genes, but did not reach the number of genes investigated when mapping *Pachycladon* tags to *P. fastigiatum* full-length ESTs. Clearly, more genes were investigated when tags were mapped against all contigs of the *P. fastigiatum* EST library or the entire TAIR10 collection. For abbreviations of different sets of reference genes see Additional file 3. **b**) **Number of differentially expressed genes (DEGs) in *P. enysii* and *P. fastigiatum* with different sets of reference genes.** The number of DEGs identified in each species is correlated with the total number of genes amenable to study for differential gene expression (compare Figure 2a and Figure 2b). The percentages of up-regulated genes in relation to the investigated gene sets ranged from 6-18% in *P. fastigiatum* and from 9-22% in *P. enysii*. Except for reference sets PL0 and AL2, more DEGs were identified in *P. enysii* than in *P. fastigiatum*.

The 20 bp tags were mapped without mismatches against 7,128 ESTs of *P. fastigiatum* representing 6,428 different gene loci (P0). 26–29% (PE) and 27–31% (PF) of all tags per lane mapped to at least one EST (Additional file 3). However, about 2% of the tags per lane were excluded from further analyses because they mapped to more than one locus (‘ambiguous tags’). This resulted in 24–27% (PE) and 26–30% (PF) unambiguous tags per lane to be analyzed for differential expression (Additional file 3). Tag counts were obtained for 6,122 *P. fastigiatum* reference genes (Figure 2a) as 163 reference genes did not contain a *Dpn*II site (Additional file 2). A further 843 reference genes, with at least one *Dpn*II site, had no tag mapping to them.

To accommodate possible SNPs between the two *Pachycladon* species we also mapped the tags of *P. enysii* with up to one mismatch to the *P. fastigiatum* references ESTs (P1). The percentage of mapped *P. enysii* tags increased from 26–29% in P0 to 33–37% in P1, with 3% of the tags mapping ambiguously (Additional file 3). Allowing for one mismatch increased the number of genes surveyed to 6,177 (Figure 2a).

Most contigs in a *de novo* assembled EST library do not represent full length transcripts. In order to test whether partial transcripts could be used as a reference for tag profiling, we mapped tags against all available contigs, first without allowing for mismatches in both species (PL0) and then with up to one mismatch in *P. enysii* (PL1). Using this approach, 16,635 and 16,906 different genes were surveyed, respectively (Figure 2a). With the PL0 approach, 64–70% (PE) and 64–75% (PF) of the tags mapped to at least one contig, and 53–58% (PE) and 54–62% (PF) mapped unambiguously. Allowing for one mismatch in the *P. enysii* tags increased the percentage of mapped tags to 73–82% and the percentage of unambiguously mapping tags to 60–71%.

Mapping with zero or one mismatch against full length transcripts or all available contigs, the gene with the highest number of tags mapping for both *Pachycladon* species was AT1G78370 (FIP1), a gene that functions in cell elongation and plant development [24]. Other genes to which a high number of tags mapped differed slightly depending on whether a mismatch was allowed and on whether full length transcripts or all available contigs were used as a reference. Using the smaller reference sets (P0 and P1), AT1G54040 (ESP) and AT3G14210 (ESM1) harboured a high number of tags in *P. enysii* and *P. fastigiatum*, respectively, while AT2G42540 (COR15A) had high counts in both species. In the P1 dataset a very high number of *P. enysii* tags also mapped to AT2G34430 (LHB1B1). Using the larger references sets (PL0 and PL1), the second and third most highly expressed genes were AT5G26000 (TGG1) and AT2G34420 (LHB1B2) in *P. enysii* and AT2G34420 (LHB1B2) and AT1G20620 (SEN2) in *P. fastigiatum*.

### Tag mapping to *A. thaliana* ESTs

The 20 bp tags were also mapped against the 6,428 orthologous genes of *A. thaliana* and all cDNA sequences of the TAIR10 database allowing for no (datasets A0, AL0), one (datasets A1, AL1) and two (datasets A2, AL2) mismatches. The results for these mappings differed considerably to the mappings against the *P. fastigiatum* ESTs (Additional file 3). If no mismatches were allowed, only about 9% (A0) or 15% (AL0) of the tags mapped to a gene of *A. thaliana.* When allowing for one mismatch and using the small dataset (A1), the percentages increased to 20-24% (*P. enysii)* and 19–23% (*P. fastigiatum*). When two mismatches were allowed (A2), the number of tags mapping was 48–63% in *P. enysii and* 49–60% in *P. fastigiatum*. When allowing for one mismatch and using the large dataset (AL1), the percentages increased to 30–34% (*P. enysii)* and 28–34% (*P. fastigiatum*). When two mismatches were allowed (AL2), the number of tags mapping was 58–65% in *P. enysii* and 55–64% in *P. fastigiatum*. Using the reduced *A. thaliana* reference set of genes, 4%, 10%, and 14% (A0, A1, A2) of the tags were available for analysis after excluding the ambiguously mapping tags. This contrasted with 10–11%, 24–28%, and 38–45% (AL0, AL1, AL2) when the entire TAIR 10 dataset was used.

When using the small *A. thaliana* dataset, the number of genes surveyed for gene expression increased with the number of allowed mismatches during mapping (from 3,884 genes in A0 to 5,233 genes in A1 to 5,490 genes in A2, Figure 2a) but did not reach the number of genes analyzed when using the small *P. fastigiatum* dataset. Using the large *Arabidopsis* dataset, 13,237 genes were surveyed in AL0, 20,273 in AL1, and 28,069 in AL2 (Figure 2a).

With the small *Arabidopsis* reference transcriptome, the most highly expressed genes differed from those found with the *P. fastigiatum* reference transcriptome and between the different datasets. In particular, with no mismatches between the reference and the tags (A0) the gene with the most tags in *P. enysii* was AT3G22840 (ELIP), while in *P. fastigiatum* it was AT1G61520 (LHCA3). When allowing for one mismatch (A1) the highest numbers of tags in both species was observed for the photosystem II protein psbW (AT2G30570). However, when allowing for two mismatches (A2), the most highly expressed gene in both species was the same as with the *P. fastigiatum* reference ESTs, namely AT1G78370 (FIP1). Tags mapping to ESP in *P. enysii* were less than a hundred in the A1 and A2 datasets and zero in the A0 dataset. Also, less than four hundred tags mapped to ESM1 in *P. fastigiatum* in the A0, A1 and A2 datasets.

Using all coding sequences of TAIR10 as a reference, the genes with the highest expression level in the AL0 dataset were AT5G24780 (VSP1) and AT3G61470 (LHCA2) for *P. enysii* and *P. fastigiatum*, respectively. When one and two mismatches were allowed, the gene with the highest expression level in both species was AT2G10330, a transposable element gene.

### Summary of results with different reference transcriptomes

With *P. fastigiatum* ESTs as a reference, many tags mapped (P: 26–37%, PL: 64–82%) while few tags (P: ~2%, PL: 10–14%) mapped ambiguously, even when mismatches were allowed in the *P. enysii t*ags. With *A. thaliana* ESTs as a reference, considerably fewer tags mapped and although the number of mapped tags increased when allowing for mismatches during mapping so did the number of ambiguous tags. For example, with the small reference set, 48–63% of all tags per lane mapped when two mismatches were allowed, but only about 14% of them mapped unambiguously and could thus be used for the differential expression analysis. With the large reference set and two mismatches allowed, the numbers improved (38–45% of all tags were used in the differential expression analysis) but did not reach the numbers obtained when using the large set of partial *P. fastigiatum* ESTs (54-71%).

The analysis of the genes with the highest expression levels did not show significant differences between the *Pachycladon* datasets but was significantly different between the *Pachycladon* and the *Arabidopsis* datasets. An investigation of the reference and tag sequences of the ESP, ESM1, and FIP1 genes revealed several explanations for this (data not shown). A deletion in the *Arabidopsis* ESP gene at the most abundant tag position led to zero counts for ESP in the datasets with no mismatch allowed. With one and two mismatches, additional tags mapping to other positions were counted. The most abundant tag in the ESM1 gene showed three SNPs between the *Pachycladon* and the *A. thaliana* sequence. Again, additional tags mapped to other positions in the *A. thaliana* reference ESM1 regardless of the number of mismatches allowed. Two mismatches and an insertion at the most abundant position in the *A. thaliana* EST led to low counts for the FIP1 gene.

In summary, using a distant reference transcriptome resulted in a) fewer tags mapping, b) some genes not being surveyed for differential expression and c) lower than expected levels of expression for genes whose most abundant mapping position was not conserved. Nevertheless, the greater size of the *Arabidopsis* transcriptome compared with the ones generated for *Pachycladon* meant that the scope of the differential gene expression analysis was much larger with the heterospecific than with the conspecific reference transcriptome.

### Differential expression analysis

Locus counts were assessed for differential expression by applying an exact test based on negative binomial distributions of count data as implemented in the R package edgeR [19]. For each gene, the log fold change (*M* value) was calculated as log2(*propE*)-log2(*propF*) with *propE* and *propF* representing the proportions of that gene in the *P. enysii* and *P. fastigiatum* tag library, respectively. The library sizes for each of the six lanes resulted from summing all locus counts for a particular lane and were different for each of the ten datasets (Additional file 3). In order to investigate the impact of a) the use of a relatively distant reference dataset and b) the use of partial contigs on the differential expression analysis we compared the amount and overlap of the differentially expressed genes (DEGs) found with each of the ten datasets summarized in Additional file 3.

### Comparison of tag profiling datasets: Numbers of DEGs

Using only full length transcripts of *P. fastigiatum* and allowing for no mismatch (P0) we inferred 1,039 and 1,239 differentially expressed genes for *P. fastigiatum* and *P. enysii*, respectively. When one mismatch was allowed in the *P. enysii* tags (P1) these numbers increased to 1,086 and 1,366 (Figure 2b). When mapping the tags without mismatches against all available *P. fastigiatum* contigs that were longer than 200 bp (PL0), representing the leaf transcriptome of this species, 2,722 and 2,702 genes were inferred to be differentially expressed (Figure 2b). Interestingly, allowing for one mismatch in *P. enysii* led to a decrease in the number of DEGs in *P. fastigiatum* (2,553) and an increase in *P. enysii* (3,126) (Figure 2b).

Using the small *Arabidopsis* reference dataset, only very few differentially expressed genes were identified. 249, 532, and 684 DEGs were inferred in *P. fastigiatum* and 395, 755, and 805 DEGs were inferred in *P. enysii* in the A0, A1, and A2 datasets, respectively (Figure 2b). When the tags were mapped against the 33,602 cDNA sequences, representing the complete *A. thaliana* transcriptome, these numbers increased to 1,009, 1,978, and 3,364 in *P. fastigiatum* and 1,219, 2,335, and 3,309 in *P. enysii* (AL0, AL1, AL2, Figure 2b). Thus in both the small and the large *A. thaliana* reference sets, the number of DEGs inferred increased with an increasing number of mismatches allowed (Figure 2b). This increase was stronger in the large *A. thaliana* reference sets (Figure 2b).

### Comparison of tag profiling datasets: Agreements and discrepancies between sets of DEGs

When comparing DEGs inferred for different datasets it is important to not only compare their number but also whether the same genes are inferred to be up-regulated between different datasets. For example, although the number of DEGs inferred with datasets P0 and P1 suggests a high degree of similarity, only 926 and 774 genes are up-regulated in both datasets for *P. enysii* and *P. fastigiatum*, respectively. We computed the number of overlapping genes in pairwise comparisons of all ten datasets (Figure 1a). For the genes up-regulated in *P. fastigiatum,* the highest overlap to the P0 dataset was 80% with PL0. The overlap of P0 with the three small *A. thaliana* datasets was higher (67–71%) than the overlap between PL0 and the large *A. thaliana* datasets (50–55%). Overall, the overlap between all *P. fastigiatum* datasets and the large *A. thaliana* datasets was only 44–55% indicating that the type of DEGs identified strongly differed depending on the reference set. The analysis of overlaps between up-regulated genes in *P. enysii* showed similar results (Figure 1a).

Not only was the overlap between the sets of up-regulated genes low for some comparisons but there were also discrepancies, i.e. cases in which one gene was inferred to be up-regulated in *P. enysii* in one dataset but in *P. fastigiatum* in the other. Although percentages of disagreements were low for most comparisons (1–6%) there were many discrepancies between the large references sets (11%, 9%, 8% between dataset PL0 and datasets AL0, AL1, and AL2, respectively, Figure 1b). Surprisingly there was also some disagreement between PL0 and PL1 (4%).

### Agreements and discrepancies between tag profiling and microarray analysis

To determine the degree of concordance between tag profiling and microarray analysis, we intersected lists of DEGs obtained with both methods and calculated confirmation percentages as above. All percentages were statistically significant irrespective of the reference set used, i.e. the overlap was higher than expected to occur by chance when intersecting lists of the respective sizes. With *P. fastigiatum* sequences as references the confirmation percentages were higher than with *A. thaliana* reference sets (49–60% vs. 21–46%). As found for the number of genes surveyed and the number of differentially expressed genes, confirmation percentages increased with an increasing number of mismatches in the *A. thaliana* datasets (Figure 1a). Interestingly, with *Pachycladon* references, confirmation percentages were slightly higher in *P. fastigiatum* than in *P. enysii* (53–60% vs. 49–51%, Figure 1a) but with *A. thaliana* references, confirmation percentages were similar in *P. fastigiatum* and *P. enysii* (23–42% and 21–46%).

In addition to cases for which microarray analyses and tag profiling identified the same DEGs, we also investigated cases for which both methods contradicted each other. To calculate disagreements we intersected ‘opposite’ lists. All intersections were not statistically significant, e. g. they were not higher than expected by chance when intersecting lists of the respective sizes. Disagreements were higher in *P. fastigiatum* than *P. enysii* across all datasets (6–14% vs. 0–7%, Figure 1b). Also, disagreements were more variable when using *A. thaliana* reference sets as opposed to using *P. fastigiatum* reference sets (0–14% vs. 5–12%, Figure 1b).

### Gene enrichment analysis of differentially expressed genes

A gene-annotation enrichment analysis (agriGO, [21]) was conducted to determine whether tag profiling analyses indicated similar ontologies as predicted from microarray analyses of the same RNA samples.

**Figure 3:**
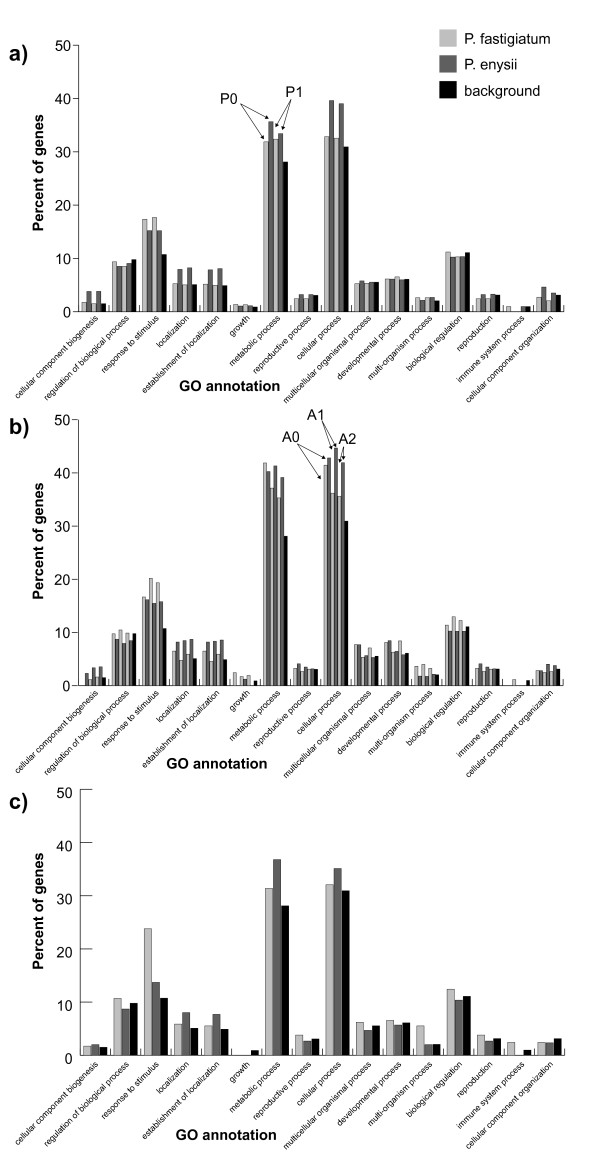
**AgriGO analyses of differentially expressed genes obtained with reference sets P0 and P1 (a), A0, A1 and A2 (b) and microarrays (c).** With microarrays, category ‘response to stimulus’ was enriched in *P. fastigiatum* and categories ‘cellular process’, ‘metabolic process’, ‘localization’ and ‘establishment of localization’ were enriched in *P. enysii* (c). The same categories were enriched with tag profiling when using the *P. fastigiatum* reference ESTs with category ‘response to stimulus’ also enriched in *P. enysii* (a). Also, similar enrichment patterns were found when using *A. thaliana* ESTs as a reference, except for the differences between species being not as clear for some categories when mapping with no mismatch (A0).

Figure 3 indicates GO term categories for which differential expression was detected when a) *P. fastigiatum* (P0, P1) and b) *A. thaliana* (A0, A1, A2) were used as a reference. Figure 3 c) shows GO terms for the microarray study [12]. In the latter, the GO term enriched in *P. fastigiatum* was ‘response to stimulus’ whereas GO terms enriched in *P. enysii* were ‘localization’, ‘establishment of localization’, ‘metabolic and cellular process’ (Figure 3c). Similar results were obtained when using the P0 and P1 datasets (Figure 3a) which in turn did not differ significantly from each other. When comparing datasets A0, A1, and A2, the results differed in some GO terms but not in a consistent way. For example, the category ‘response to stimulus’ was equally enriched in *P. enysii* and *P. fastigiatum* in the A0 dataset but more highly enriched in *P. fastigiatum* in the A1 and A2 datasets. For the GO terms ‘metabolic process’ and ‘cellular process’ enrichment percentages were decreasing with the number of mismatches in *P. fastigiatum*. Similar to the microarray analysis they were higher in *P. enysii* except for the enrichment percentage for ‘metabolic process’, which was higher in *P. fastigiatum* with A0.

Annotations of *A. thaliana* for the 1,039 and 1,239 up-regulated genes of *P. fastigiatum* and *P. enysii*, respectively (P0) were analysed using DAVID [22,23] against the curated GO database for biological processes (GOTERM_BP_FAT) and the KEGG reference database. GO annotations were found for 3,336 of the 6,428 reference loci that were used as a population background and for 562 and 708 of the 1,039 and 1,239 up-regulated genes. Seven clusters in *P. fastigiatum* and one in *P. enysii*, had a significant enrichment score greater than 1.3 or contained GO terms with a *p*-value smaller than 0.05 (Additional file 4). The cluster with the highest enrichment score (1.63) in *P. fastigiatum* contained genes belonging to GO terms associated with ‘regulation of transcription’, while the second highest scoring cluster contained genes for the GO term ‘response to water deprivation’ which was also enriched in the microarray analysis. These genes included ERD7 and ERD10 (genes showing early response to water deprivation: AT2G17840 and AT1G20450) as well as the genes RD2 and RD20 (genes responsive to desiccation: AT2G21620 and AT2G33380). Another cluster with a significant enrichment score (1.41) harboured genes associated with ‘defense response to fungus’. Other clusters with significant GO terms were associated with ‘nucleoside metabolic process‘(1.21), ‘response to fungus‘(1.21), ‘response to hydrogen peroxide‘(0.95), and ‘response to oxidative stress‘(0.86). In *P. enysii* the only cluster with an enrichment score higher than 1.3 (2.39) contained GO terms associated with ‘macromolecular complex subunit organisation‘.

In summary, *P. fastigiatum* was most significantly enriched for GO terms associated with stress responses in various forms (fungus, water, oxidation) while no such GO terms were found enriched in *P. enysii*.

**Figure 4:**
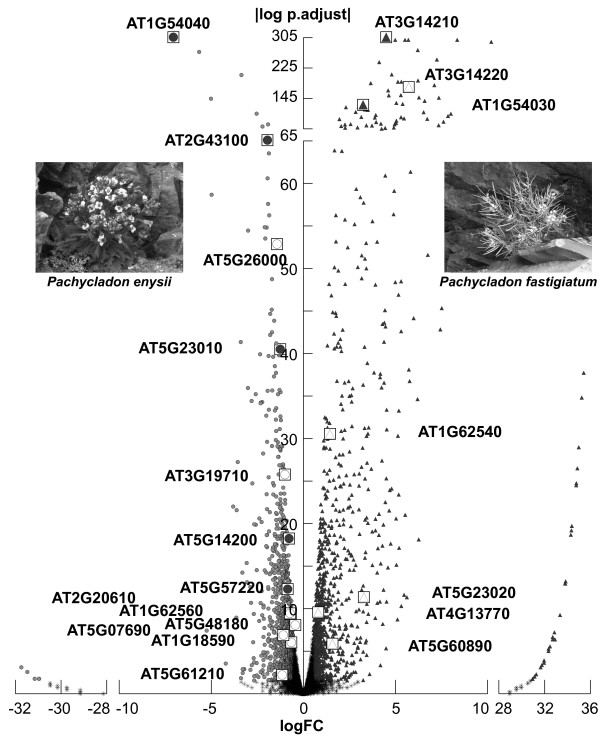
**Volcano plot depicting DEGs obtained using *P. fastigiatum* full-lengths ESTs as a reference for tag mapping (P0).** 1,239 and 1,039 genes were determined as differentially expressed in *P. enysii* (circles) and *P. fastigiatum* (triangles), respectively. Log fold ratios >28 and < −28 indicate genes with zero tags in either of both species whereas log fold ratios between −10 and 10 indicates genes with tags present in both species but differing in abundance. Five glucosinolate metabolism loci show similar up-regulation as in a previous microarray analysis [12] in *P. enysii* (AT1G54040, AT2G43100, AT5G23010, AT5G14200, AT5G57220; filled circles) as well as two loci in *P. fastigiatum* (AT3G14210, AT1G54030; filled triangles). Thus conclusions drawn from microarray analyses regarding glucosinolate phenotypes (AT1G54040 - ESP up-regulation indicates that *P. enysii* produces nitriles; AT3G14210 - ESM1 up-regulation indicates that *P. fastigiatum* produces isothiocyanates; AT5G23010, AT2G43100, AT5G14200 - up-regulation of methylthioalkylmalate synthase 1, methylthioalkylmalate isomerase, methylthioalkylmalate dehydrogenase indicates that *P. enysii* produces C4 glucosinolates) can be equally drawn from this tag profiling study. Eight additional loci involved in glucosinolate metabolism could be identified for *P. enysii* (AT5G26000, AT3G19710, AT2G20610, AT1G62560, AT5G48180, AT1G18590, AT5G07690, AT5G61210; empty circles) and five for *P. fastigiatum* (AT3G14220, AT1G62540, AT5G23020, AT4G13770, AT5G60890; empty triangles).

Further insight concerning differences in support for ontology inferences can best be gained and illustrated by reference to specific examples (Figure 4, Additional file 5).

#### Glucosinolate metabolism

Glucosinolates and their hydrolysis products have been implicated in defense against herbivores and pathogens. Nine marker genes for glucosinolate metabolism that were differentially expressed in our previous microarray study were found to have identical expression patterns in most tag profiling datasets (Figure 4). These include the ESM1 gene, a marker for isothiocyanate production (AT3G14210, [25,26]) which was inferred to be up-regulated in *P. fastigiatum* in all ten datasets. The MVP1 gene (AT1G54030, [27]) a myrosinase associated protein specifically interacting with TGG2, was also up-regulated in *P. fastigiatum* in all datasets except A0 and AL0. The ESP gene (AT1G54040), a marker for nitrile production [28] as well as two marker genes for the production of methionine-derived glucosinolates with four carbon atoms (methylthioalkylmalate isomerase, IPMI SSU2, AT2G43100; cytochrome P450, CYP81F2, AT5G57220; [29,30]) were up-regulated in *P. enysii* in all datasets except A0 and AL0 and two other marker genes for C4 glucosinolates (methylthioalkylmalate synthase 1, MAM1, AT5G23010; methylthioalkylmalate dehydrogenase, IPMDH1, AT5G14200) were up-regulated in *P. enysii* in all datasets. AT1G74100 (SOT16) was up-regulated in *P. enysii* in the PL0 dataset and AT4G03060 (AOP2) was up-regulated in *P. enysii* in the AL1 and AL2 datasets.

Both microarray analyses and tag profiling identified differentially expressed glucosinolate metabolism genes not observed with the other method (Figure 4). With tag profiling five additional genes were inferred to be up-regulated in *P. fastigiatum* (AT3G14220 (GDL20; except A0 and AL0), AT1G62540 (FMO-GSOX2, conversion of methylthioalkyl glucosinolates to methylsulfinylalkyl glucosinolates, [31]; all *Pachycladon* datasets), AT4G13770 (REF2, conversion of aldoximes to thiohydroxymates, [32,33]; except A0, A1, AL0, AL1), AT5G23020 (MAM3, methylthioalkylmalate synthase involved in C3-C8 glucosinolate biosynthesis, [34]; P0, PL0, A2, AL1), AT5G60890 (MYB34, control of indolic glucosinolate homeostasis, [35]; all *Pachycladon* datasets)). With tag profiling twelve additional genes were inferred to be up-regulated in *P. enysii* (AT1G18590 (SOT17; except AL0, AL1, AL2), AT1G62560 (FMO-GSOX3; all datasets), AT2G20610 (SUR1; except A0 and AL0), AT3G19710 (BCAT4; except A0), AT5G26000 (TGG1; except A0, AL0, AL1), AT5G48180 (NSP5; A0, A1, P0, AL0); AT5G61420 (MYB28; all *Arabidopsis* datasets), AT5G07690 (MYB29; except A0, A1, AL0), AT5G25980 (TGG2; PL1, AL0, AL1, AL2), AT5G61210 (SNP33; P0, P1), AT1G04750 (VAMP721; AL0, AL1, AL2), AT1G59870 (PEN3; AL1), [28,29,31]).

The homologs to AT1G54000 (GLL22, [27]), AT1G31180 (IPMDH3), AT2G14750 (APK1), AT3G58990 (IPMI-SSU3), and AT4G03050 (AOP3) [31] were up-regulated in *P. enysii* in the microarray analysis only. Contradictory results were obtained for the homologue to AT1G54020, a myrosinase-associated protein closely related to ESM1 and MVP1, as it was up-regulated in *P. fastigiatum* in the microarray analysis but in *P. enysii* when measured by tag profiling.

#### Response to cold

Populations of *P. fastigiatum* grow at a mean altitude of 1,485 m, while *P. enysii* grows between heights of 1,476 and 2,492 m [11]. Both plants, but *P. enysii* much more so, are subject to cold temperatures. An enrichment of GO terms corresponding to cold stimulus was detected in the microarray experiment *for P. fastigiatum*[12]. While this GO term was not enriched with tag profiling, two genes involved in cold tolerance in *A. thaliana* (AT5G66400 (RAB18) and AT1G20440 (COR47) [36]) were up-regulated in *P. fastigiatum* in all tag profiling datasets. Up-regulation of AT1G20440 was also detected in *P. fastigiatum* with the microarray. Other genes related to cold tolerance and inferred to be up-regulated in most, but not all tag profiling datasets were AT1G20450 (ERD10; except A0-2), AT1G04400 (CRY; except P1, PL1), AT2G45660 (SOC1, except P1, PL1), AT4G22950 (AGL19, except P0, A1, A2), AT2G33835 (FES1; only AL2, PL0), and AT4G25140 (OLE1; only AL2) in *P. fastigiatum* and AT4G25530 (FWA, only PL0), AT2G19520 (FVE; AL0), and AT1G31812 (ACBP; all) in *P. enysii.* The gene XERO2 (AT3G50970) was only found up-regulated in *P. fastigiatum* in the microarray analysis but not with tag profiling. These results suggest that, although genes implicated in cold tolerance were up-regulated in both species, the response to cold was more substantial in *P. fastigiatum*.

#### Flower development

A process not detected from differential expression or ontology analyses of the heterologous microarrays, but detected by tag profiling was flower induction. AT4G31120 (SKB1), a gene that promotes flowering by repressing flowering locus C (FLC, AT5G10140) [37] was up-regulated in *P. enysii* in all datasets except A0 and A1. FLC was up-regulated in *P. fastigiatum* in all datasets except A0 and AL0. Another gene known to be a repressor of flowering locus C, AT3G18990 (VRN1) was also up-regulated in *P. enysii* in datasets PL0 and PL1. Because of their higher altitude, *P. enysii* populations are covered with snow for the most part of the year leaving a relatively short timeframe to develop flowers and seeds. Although plants for both species were collected at the same time of year, and prior to flowering, up-regulation of genes that induce flowering in *P. enysii* may be indicative of an earlier flowering time for this species.

### Analysis of homeologous gene copies

**Figure 5:**
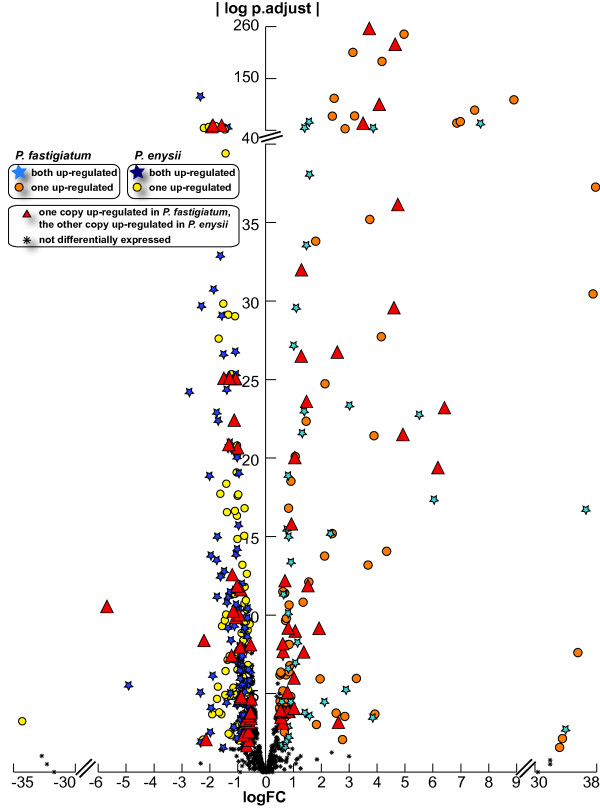
**Volcano plot depicting differentially expressed homeologous gene copies obtained using *P. fastigiatum* full-lengths ESTs as a reference for tag mapping.** With gene loci for which gene expression of individual homeologous copies could be studied we identified 245 genes up-regulated in *P. enysii* and 136 genes up-regulated in *P. fastigiatum.* 392 such loci were not differentially expressed between species (black cross). Among the up-regulated genes, six different patterns were detected. In 111 cases all copies of one locus were up-regulated in *P. enysii (*dark blue stars); in 38 cases both copies were up-regulated in *P. fastigiatum* (light blue stars); for 101 and 65 sequences, one copy was up-regulated in *P. enysii* (yellow circles) and *P. fastigiatum* (orange circles)*,* respectively, whereas the other copy was not differentially expressed and in 66 cases one copy was up-regulated in one species while the other was up-regulated in the other (red triangles). Log fold ratios >30 and < −30 indicate genes with zero tags in either of both species whereas log fold ratios between −10 and 10 indicate genes with tags present in both species but differing in abundance.

We investigated 379 homeologous gene pairs and five gene triplets consisting of two homeologues and one paralogue for copy-specific expression. Of these 773 genes, 245 and 136 were up-regulated in *P. enysii* and *P. fastigiatum,* respectively, whereas 392 copies were not differentially expressed. For 55 and 19 pairs, both copies were up-regulated in *P. enysii* and *P. fastigiatum,* respectively. For 101 and 64 pairs, one copy was up-regulated in *P. enysii* and *P. fastigiatum,* respectively, whereas the other copy was not differentially expressed. For 33 pairs one copy was up-regulated in one species while the other was up-regulated in the other species (Figure 5, Additional file 5).

Myrosinase-associated proteins (MyAP) which function in glucosinolate metabolism had a variable number of homeologous copies in the *P. fastigiatum* EST library: none for AT1G54000 and AT1G54010, one for AT1G54020, AT3G14220, and AT3G14225 (GLIP4), and three for AT1G54030 (MVP1). Two of the MVP1 sequences are most similar to a homologue on chromosome 1 in *A. thaliana* but the third MVP1 sequence is most similar to a gene in the *Arabidopsis lyrata* genome that precedes the ESM1 gene on chromosome 3. Differential expression for some of the MyAP copies was observed. AT1G54020 was up-regulated in *P. enysii* whereas one of the homeologous MVP1 was up-regulated in *P. fastigiatum*. The highest number of tags mapped to the third MVP1 copy that was also up-regulated in *P. fastigiatum*. Myrosinase TGG2, which is known to interact with MVP1 [27], was not present in the *P. fastigiatum* EST library. However, one copy of myrosinase TGG1 was up-regulated in *P. enysii*.

For four *A. thaliana* genes - AT1G52740 (HTA9), AT3G53730, AT1G51650, AT3G15450 - genes, three homologous sequences had been assembled in the *P. fastigiatum* EST library. All three sequences homologous to AT1G52740, two sequences homologous to AT3G53730 and one sequence homologous to AT1G51650 were up-regulated in *P. enysii*. Of the AT3G15450 homologs, one was up-regulated in *P. enysii*, one was up-regulated in *P. fastigiatum* and the third was not differentially expressed.

## Discussion

### Tag profiling as a method for expression profiling

Tag profiling as a means to study differential gene expression [8,38] has been successfully applied in bats [39], maize [40], planthoppers [41], honey bees [42] and mice [5]. However, tag profiling is only one of a number of alternative approaches for expression profiling. Microarrays have been the gold standard in the past and more recently RNA-seq has gained increasing popularity [43]. Our study of 20mer tag profiles for two closely related NZ alpine Brassicaceae – *Pachycladon enysii* and *Pachycladon fastigiatum* – suggests that not only is tag sequencing superior to microarray analyses, but in some cases can be expected to have significant advantages over RNA-seq.

As previously stated there are several shortcomings to microarray technologies [5]. With hybridization-based methods, genes with low expression levels cannot be analysed whereas with sequencing-based methods even absence of expression can be distinguished from low expression and expression can be detected over several orders of magnitude. This much greater dynamic range of sequencing approaches over microarrays has led to predictions that the former will eventually replace the latter [44]. Consistent with a higher dynamic range, we observed more differentially expressed genes with tag profiling (6–22% depending on reference set) than with the earlier heterologous microarray study (~2%, [12]).

One limitation of the tag sequencing protocol used in the present study is the dependence on the presence of a *Dpn*II or *Nla*III restriction site in the transcript. Thus 2.3% of our reference genes were unavailable for analysis. This limitation and others, such as ease in multiplexing samples, are reasons that some researchers are pursuing other tag sequencing protocols such as CAGE and SuperSAGE [7,45]. Irrespective of the best protocol used for generating short sequence tags, our study provides insight into the potential of short tag sequencing as a method for investigating and inferring differential expression when different reference transcriptomes are available.

In our current work we are interested in studying plant responses to environmental variation - studies that require a large number of sample comparisons. Hence finding a reliable and cost effective profiling method is important. Although high throughput sequencing is becoming more affordable, tag sequencing has cost advantages over RNA-seq analyses. The sequencing of 20–30 bp tags provides much greater sequencing depth and also decreases the complexity of the differential expression analysis compared to analyses based on random 75–150 bp RNA-seq reads [45]. For example, the statistics needed to analyse RNA-seq experiments are known to introduce a length bias, with longer genes having a higher probability of being inferred to be differentially expressed [46]. This problem does not affect tag sequencing.

However, are 20mer sequence tags sufficient in length for purposes such as we are interested? Previously it has been stated that 20mer sequence tags cannot be effectively used for profiling species when a same species reference transcriptome is not available [45]. Our results do not support this conclusion. As we discuss in the following section, the choice of reference transcriptome and mapping parameters have important implications for biological inferences.

### The choice of a reference transcriptome

Developing tag-based approaches to gene expression profiling in a new species or group of closely related species requires consideration to be given to what type of reference library is being used. It is not clear a) if a heterospecific but complete and well annotated transcriptome can serve as a reference, b) how much information is lost by using such a distant reference, c) how using a less well developed but conspecific reference library compares to using a heterospecific library and d) what mapping parameters should be used in both cases. We addressed all of these aspects in our study.

For mapping we used four different reference transcriptomes: 1) an EST library of 6,428 full-length ESTs of *Pachycladon fastigiatum* leaf tissue, 2) orthologous cDNA sequences from *Arabidopsis thaliana*, 3) all partial contigs of *P. fastigiatum* that have been assembled from 75 bp reads in our lab, and 4) all transcripts available in the TAIR10 database. Given that *Pachycladon* is an allopolyploid genus [10], we expected to find two copies from different parental genomes (homeologous copies) for many genes. 700 homeologous pairs were represented among the full lengths cDNAs in our EST library. *A. thaliana* and *P. fastigiatum* reference ESTs were on average 90% identical. While homeologous copies within one *Pachycladon* species had about 90% identical sites, the respective orthologous genes in different species, e.g. *P. fastigiatum* and *P. cheesemanii,* were up to 98% identical [13]. Therefore we were optimistic to not only be able to map *P. enysii* tags to *P. fastigiatum* ESTs but also to acquire different tag counts for the homeologous copies of some genes for both species. Mapping tags to *P. fastigiatum* full length sequences was in many ways superior to mapping tags to the orthologous *A. thaliana* transcripts. Less data were lost (34–36% of the tags mapped to *P. fastigiatum* ESTs while only 10% of the tags mapped to *A. thaliana* transcripts with no mismatches). Less tags mapped ambiguously (28–31% of the tags mapped to unique *P. fastigiatum* ESTs while only 4–14% tags mapped to unique *A. thaliana* ESTs). More genes could be analyzed for differential expression (6,122 genes with the *P. fastigiatum* reference while only 3,884 genes could be studied with the *A. thaliana* reference when mapping with no mismatches). More differentially expressed genes were found (e.g. 1,239 genes were identified as up-regulated genes in *P. enysii* with the *P. fastigiatum* reference while 394 were identified as up-regulated genes in *P. enysii* with the *A. thaliana* reference when mapping with no mismatches) and previous microarray results were more clearly confirmed (46–63% vs 28–44%).

Increasing the number of mismatches between *Pachycladon* tags and *A. thaliana* transcripts had positive as well as negative consequences; the percentage of mapped tags increased but so did the number of ambiguous mappings. Also, the number of genes surveyed increased although not up to the number used in the analysis of *P. fastigiatum* full lengths ESTs. When mapping against the distant reference, some tag positions were lost and thus these did not contribute to the total tag count for a gene. For example, because the number of SNPs in the most abundant tag position of the ESM1 gene exceeded the number of mismatches allowed, expression levels for ESM1 were wrongly detected as being very low although the gene was still identified as differentially expressed in *P. fastigiatum*. Similarly for ESP, the most abundant tag position was not counted because of a deletion in the *A. thaliana* ortholog. However, despite an underestimation of expression for ESP, differential expression in *P. enysii* was still detected due to other low abundant tag positions mapping to the *A. thaliana* ortholog. Both, ESP and ESM1 are markers for adaptive phenotypes [26,47].

Mapping against the entire collection of *P. fastigiatum* ESTs (full-length plus partial) was successful as long as the partial contigs had a restriction site and were reliably annotated. Although the detection of differential expression was possible, gene expression levels may have been underestimated as tag counts may have been incomplete. Care was taken in that reads mapping to overlapping contigs of the same gene were not counted twice. By not restricting our analysis to full-length ESTs, the number of genes amenable to study increased as did the number of DEGs. For example, we were able to measure differential expression of the glucosinolate metabolism gene SOC16 (AT1G74100) and the repressor of flowering locus C (AT3G18990) in *P. enysii*, two genes potentially involved in adaptive processes.

When extending our analysis to the complete collection of *A. thaliana* gene models, we were able to monitor even more genes for differential expression than with all *P. fastigiatum* reference ESTs. Also amongst those additional genes were genes of potential adaptive significance as the AOP2 gene which we expected to be up-regulated in *P. enysii* from our previous microarray analysis but which did not assemble in our *P. fastigiatum* reference library. Only with the large *A. thaliana* reference sets and allowing for one or two mismatches, this gene was correctly identified as being differentially expressed in *P. enysii*. However, as was the case for the small *A. thaliana* reference sets, with an increasing number of mismatches, the number of ambiguously mapping tags increased.

Taken together, our findings demonstrate that the construction of a reference transcriptome for the focal species (or a close relative thereof) is preferable to using a reference transcriptome with 90% similarity to the focal species. In particular, if the goal is to identify genes involved in adaptive processes, a conspecific reference transcriptome is desirable as these genes often evolve sequence differences between species (see above discussion on ESM1 and ESP). Partial conspecific reference sequences should be included as additional insights can be gained. However, if it is necessary to use a heterospecific reference transcriptome, our experience suggests that it is important that mapping parameters (such as number of mismatches) are optimized to maximize both the scope (e.g. percentage of mapped tags and genes surveyed) and reliability (e.g. number of ambiguous mappings) of the analysis. Wang et al. mapped tags derived from bat mRNA to well annotated mouse and human references (less than 90% similarity) [39]. This approach while successful and informative, would have limited their analysis to genes conserved between the reference and species of interest excluding for example those genes that are present only in the analysed species due to a higher ploidy level or to recent duplications of single genes.

### Does tag profiling provide more biological insights than microarrays?

Our gene ontology analysis of tag profiles revealed similar major GO terms to be enriched in *P. enysii* and *P. fastigiatum* as with microarray-derived expression profiles [12]. Finer resolution GO analyses also identified similar enriched GO terms between both platforms. Most notably these were stress response GO terms such as response to dessication/water deprivation and response to oxidation in *P. fastigiatum*.

Since both analyses differed in scope - the microarray analysis gave results for 18,094 loci while only 6,121 different gene loci were included in the EST library of *P. fastigiatum* and were hit by at least one tag – comparisons were possible for 4,969 loci. Twentyone to 60% of the genes up-regulated in the microarray analyses were also up-regulated in the tag profiling analysis with percentages varying with different reference gene sets. We also detected a low level of disagreement between tag profiling and microarray results (0–14%) but in contrast to all agreements that were statistically significant, the disagreements did not exceed those expected to occur by chance.

To further compare inferences from both gene expression technologies we investigated the expression of genes involved in glucosinolate metabolism, cold tolerance and flowering as these are traits of potential adaptive significance in the divergence of both species*.* Conclusions of biological significance, namely, the difference in glucosinolate hydrolysis products (*P. enysii* produces nitriles, *P. fastigiatum* produces isothiocyanates) and chain length of glucosinolates (*P. enysii* produces C4 whereas *P. fastigiatum* produces C3), which had been predicted by the differential expression of underlying genes in the microarray analysis, could also be drawn from our tag profiling studies as similar gene expression patterns were found. In addition to those confirmed genes, with tag profiling another set of glucosinolate metabolism genes was inferred to be differentially expressed. Another chain elongation locus (BCAT4) and nitrile specifier (NSP5) were up-regulated in *P. enysii* supporting the prediction of C4 glucosinolate production and nitrile formation. Interestingly, tag profiling results predict *P. enysii* and *P. fastigiatum* to use different flavin-monooxygenases to catalyze the conversion of methylthioalkyl glucosinolates to methylsulfinylalkyl glucosinolates (FMO GS-OX3 vs FMO GS-OX2). Other interesting findings by tag profiling include the up-regulation of REF2, which links phenylpropanoid and glucosinolate metabolism, and of MAM3, which mediates the synthesis of long-chain methionine glucosinolates in *P. fastigiatum* and the up-regulation of two other loci of the glucosinolate core pathway (SOT17, C-S lyase) and a myrosinase (TTG1) in *P. enysii*. Moreover, a suite of glucosinolate metabolism genes involved in fungal defense (MYB28, MYB29, TGG2, SNP33, VAMP721 and PEN3) were inferred to be up-regulated with tag profiling in *P. enysii.* Thus the earlier findings of a significantly different defence response between *P. fastigiatum* and *P. enysii* were corroborated by the tag profiling analysis. Due to the higher number of differentially expressed genes found in *P. enysii* with tag profiling the up-regulation of the glucosinolate pathway becomes more obvious. Similarly, a greater number of genes involved in cold tolerance were differentially expressed with tag profiling as compared to microarrays. The differential expression of flowering genes had not been detected with the microarrays and may indicate different onsets of flowering in both species.

Another advantage of tag profiling over microarrays was the surveillance of homeologous copies for differential expression by computational analysis alone. A few microarray and EST library studies have attempted the distinct quantification of homeologous copies, most notably with cotton [48] and coffee [49]. However with the microarray studies, copy-specific probes had to be designed prior to the expression analysis which is not necessary with tag profiling. In our study this is best illustrated with locus AT1G54030 which was up-regulated in *P. fastigiatum* with the heterologous microarray. With tag profiling we observed that this up-regulation is due to up-regulation in one of the two homeologous copies but not both. Moreover, we discovered a third copy of the gene, most probably a paralogue on a different chromosome, to be up-regulated in *P. fastigiatum*.

With tag profiling, sequence information is obtained alongside with expression levels allowing for a high resolution analysis that renders tag profiling preferable to heterologous microarrays, particularly when studying a non-model organism with no prior sequence information. Although measuring DGE of homeologous copies was more complicated (because some tags map to both homeologous copies), we were able to make inferences for 384 of the 700 gene loci. While 196 pairs showed no differential expression between species, in other cases one copy was found to be preferentially expressed over the other. Both homeologous copies were up-regulated for 19 and 55 loci In *P. fastigiatum* and *P. enysii*, respectively. While for 64 and 101 loci, one homeologous gene copy only was up-regulated in *P. fastigiatum* and *P. enysii*, respectively. In these cases the other homeologous copy was not differentially expressed. We also detected 33 cases with one copy up-regulated in *P. fastigiatum* and the other in *P. enysii*. These cases will be subject to further analyses as for most of them no appropriate annotation could be found and thus no conclusion could be drawn about their biological significance.

In summary, biological insights obtained with tag profiling were greater as more genes of potential adaptive significance were found to be differentially expressed than with microarrays. In addition, tag profiling allowed for the analysis of differential expression of many homeologues which was not possible with the heterologous microarrays.

## Conclusions

Compared with our findings from an earlier heterologous microarray analysis, tag profiling with 20mer tags offered higher resolution, higher sensitivity, higher dynamic range and the opportunity to study differential expression of homeologues in a non-model species. When pioneering expression studies in a new species, we recommend investing in the construction of an EST library that can serve as a reference transcriptome for mapping tags as opposed to using a distant reference transcriptome. Here we demonstrated that once the reference EST library is in place, tag profiling can be effectively implemented for identifying candidate genes potentially important in biotic and abiotic interactions of non-model plants. RNA-seq studies should be considered complementary to tag sequencing protocols. Although they are not as cheap and do not offer as great a depth of coverage as tag profiling, they are likely to provide further insights into studies such as the one undertaken here. In particular the increased read length with RNA-seq means it should be easier to distinguish splice variants, homeologues and paralogs, including those that show divergence at the 3 prime end of their sequences.

## Competing interests

The authors declare that they have no competing interests.

## Authors’ contributions

CV participated in the design of the tag profiling analysis, collected the plants and extracted the RNA, participated in the differential expression analysis and drafted the manuscript. NG participated in the design of the study, the differential expression analysis, the design of the figures and participated in drafting the manuscript. PB participated in the design of the differential expression analysis and conducted the statistical test regarding the comparison between the tag profiling and microarray analysis. OD participated in the design of the tag profiling analysis, the differential expression analysis and participated in drafting the manuscript. PL participated in the design of the tag profiling analysis and in drafting the manuscript. All authors read and approved the final manuscript.

## Supplementary Material

Additional file 1 **Table S1.** Sequences and annotations for 7,128 ESTs from *Pachycladon fastigiatum***.** Click here for file

Additional file 2 **Figure S1.** In-silico distribution of GATC positions. The number of GATC positions (*Dpn*II sites) per EST of *P. fastigiatum* (black bars) and their *A. thaliana* homologs (grey bars) was determined. For 144 ESTs of *P. fastigiatum* no GATC restriction site could be found as well as for 301 genes from *A. thaliana* while there were 19 and six sequences with more than 20 restriction sites. Click here for file

Additional file 3 **Table S2.** The number of mapped and filtered reads per lane and dataset. The total number of reads for the three lanes of *P. enysii* (PE1, PE2, PE3) and *P. fastigiatum* (PF1, PF2, PF3) was determined as well as the number of reads after trimming. For the different mapping strategies, the number and percentage of reads that mapped to the reference genes and the number of tags used in the differential expression analysis are shown. Percentages are given with respect to the total number of trimmed reads. Click here for file

Additional file 4 **Table S3.** Significantly enriched clusters and GO terms of 1,039 and 1,239 loci up-regulated in *P. fastigiatum* and *P. enysii*, respectively, identified with DAVID. The 6,428 reference loci were used as population background. Clusters are ordered by enrichment score. Click here for file

Additional file 5 **Table S4.** Differential expression statistics Sheet A) Differential expression statistics for 57 and 79 genes identified as commonly up-regulated in microarray and tag profiling analyses (P0) in *P. enysii* and *P. fastigiatum,* respectively. Sheet B) Differential expression statistics for 6 and 9 genes identified as oppositely up-regulated in microarray and tag profiling analyses (P0) in *P. enysii* and *P. fastigiatum*, respectively. Sheet C) Differential expression statistics of the analysis between homeologous copies. Homeologous gene copies were analysed for differential expression at 773 gene loci. For these genes (i) both homeologous copies were present in the EST reference library and (ii) copy-specific tags could be obtained and exceeded copy-unspecific tags in abundance by at least fivefold. Sheet D) Differential expression statistics for the 1,239 and 1,039 genes up-regulated in *P. enysii* and *P. fastigiatum*, respectively in the P0 data set. Click here for file
