# Supplementary figures and images for: Chips and tags suggest plant-environment interactions differ for two alpine Pachycladon species
Source: BMC Genomics. 2012 Jul 19;13:322. doi: 10.1186/1471-2164-13-322 (PMC3460751; doi:10.1186/1471-2164-13-322)

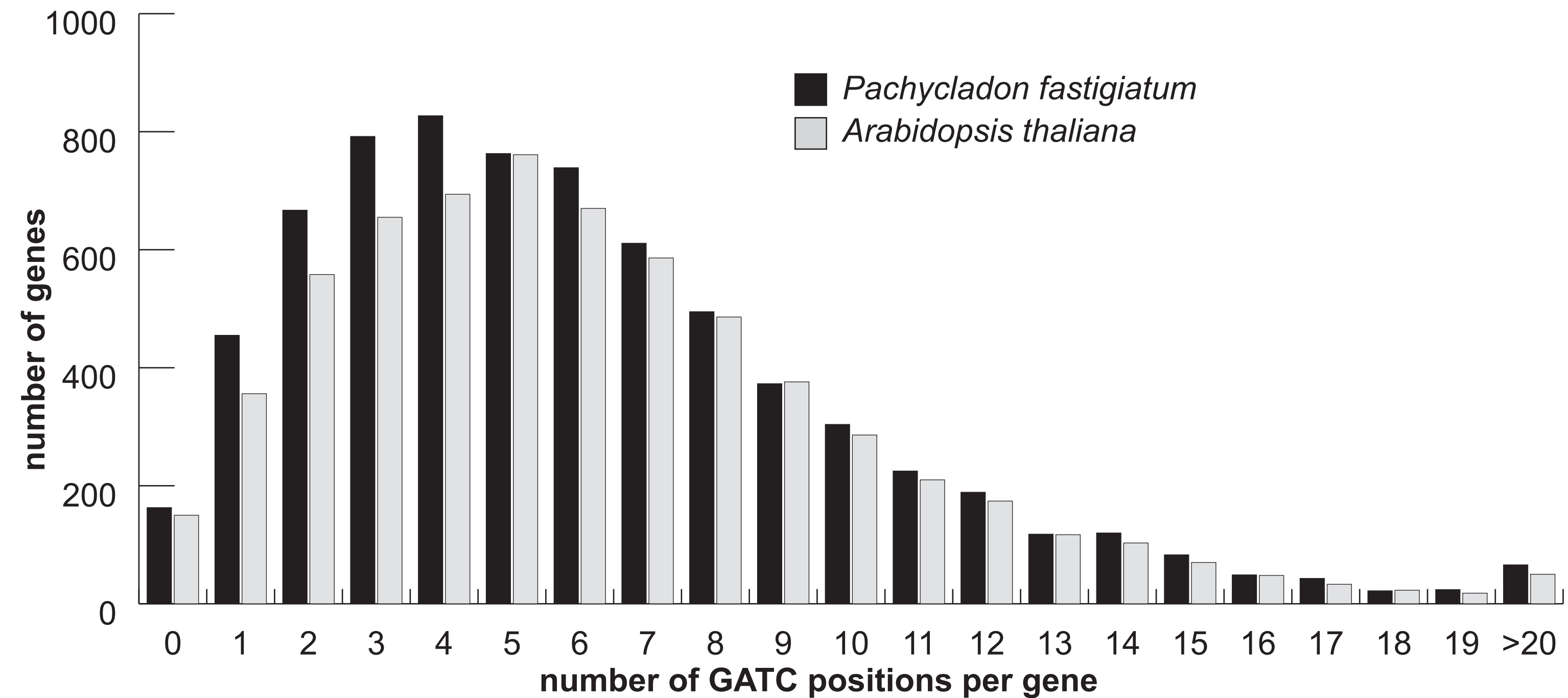

Supplement: Additional file 2 — Figure S1. In-silico distribution of GATC positions. The number of GATC positions (DpnII sites) per EST of P. fastigiatum (black bars) and their A. thaliana homologs (grey bars) was determined. For 144 ESTs of P. fastigiatum no GATC restriction site could be found as well as for 301 genes from A. thaliana while there were 19 and six sequences with more than 20 restriction sites. [file 1471-2164-13-322-S2.pdf]
