# Supplementary material for: Chips and tags suggest plant-environment interactions differ for two alpine Pachycladon species
Source: BMC Genomics. 2012 Jul 19;13:322. doi: 10.1186/1471-2164-13-322 (PMC3460751; doi:10.1186/1471-2164-13-322)
Supplement: Additional file 3 — Table S2. The number of mapped and filtered reads per lane and dataset. The total number of reads for the three lanes of P. enysii (PE1, PE2, PE3) and P. fastigiatum (PF1, PF2, PF3) was determined as well as the number of reads after trimming. For the different mapping strategies, the number and percentage of reads that mapped to the reference genes and the number of tags used in the differential expression analysis are shown. Percentages are given with respect to the total number of trimmed reads. [file 1471-2164-13-322-S3.pdf]

		PE1	PE2	PE3	PF1	PF2	PF3
total reads		3,174,313	5,514,315	6,271,593	3,792,072	5,663,506	6,456,698
reads after trimming		2,918,364 (91.94 %)	5,056,782 (91.70 %)	5,179,113 (82.58 %)	3,604,271 (95.05 %)	5,147,352 (90.89 %)	5,258,588 (81.44 %)
mapped reads to *P. fastigiatum* ESTs (**P0**)	mapping with no mismatch against 7,128 full length transcripts of *P. fastigiatum*	911,106 (28.70 %)	1,578,248 (28.62 %)	1,627,713 (25.95 %)	1,191,836 (31.43 %)	1,716,001 (30.30 %)	1,759,387 (27.25 %)
reads used in **P0**		841,651 (26.51 %)	1,457,950 (26.44 %)	1,503,077 (23.97 %)	1,124,561 (29.65 %)	1,618,794 (28.58 %)	1,660,468 (25.72 %)
mapped reads to *P. fastigiatum* ESTs (**P1**)	mapping with one (*P. enysii*) and no (*P. fastigiatum*) mismatch against 7,128 full length transcripts of *P. fastigiatum*	1,182,889 (37.26 %)	2,035,110 (36.91 %)	2,068,409 (32.98 %)	1,191,836 (31.43 %)	1,716,001 (30.30 %)	1,759,387 (27.25 %)
reads used in **P1**		1,087,143 (34.25 %)	1,873,929 (33.98 %)	1,907,873 (30.42 %)	1,124,561 (29.65 %)	1,618,794 (28.58 %)	1,660,468 (25.72 %)
mapped reads to *P. fastigiatum* ESTs (**PL0**)	mapping with no mismatch against all 9,636,919 contigs of *P. fastigiatum* library	2,225,948 (70.12 %)	3,864,943 (70.09 %)	3,991,942 (63.65 %)	2,819,316 (74.35 %)	4,062,242 (71.73 %)	4,179,345 (64.73 %)

		PE1	PE2	PE3	PF1	PF2	PF3
reads used in **PL0**		1,856,764 (58.49 %)	3,225,290 (58.49 %)	3,331,371 (53.11 %)	2,356,907 (62.15 %)	3,393,971 (59.93 %)	3,490,999 (54.07 %)
mapped reads to *P. fastigiatum* ESTs (**PL1**)	mapping with one (*P. enysii*) and no (*P. fastigiatum*) mismatch against all 9,636,919 contigs of *P. fastigiatum* library	2,602,991 (82.00 %)	4,502,193 (81.65 %)	4,606,285 (73.45)	2,819,316 (74.35 %)	4,062,242 (71.73 %)	4,179,345 (64.73 %)
reads used in **PL1**		2,256,283 (71.08 %)	3,696,537 (67.04 %)	3,791,593 (60.46 %)	2,356,907 (62.15 %)	3,393,971 (59.93 %)	3,490,999 (54.07 %)
mapped reads to *A. thaliana* ESTs (**A0**)	mapping with no mismatch against 6,428 orthologues of *P. fastigiatum* full length transcripts from *A. thaliana*	300,155 (9.46 %)	516,478 (9.37 %)	530,152 (8.45 %)	339,998 (8.97 %)	488,819 (8.63 %)	497,919 (7.71 %)
mapped reads to *A. thaliana* ESTs (**A1**)	mapping with one mismatch against 6,428 orthologues of *P. fastigiatum* full length transcripts from *A. thaliana*	747,280 (23.54 %)	1,249,054 (22.65 %)	1,233,528 (19.69 %)	853,666 (22.51 %)	1,179,212 (20.82 %)	1,209,709 (18.74 %)
mapped reads to *A. thaliana* ESTs (**A2**)	mapping with two mismatches against 6,428 orthologues of *P. fastigiatum* full length transcripts from *A. thaliana*	2,015,271 (63.49 %)	3,029,446 (54.94 %)	3,029,446 (48.30 %)	2,291,344 (60.42 %)	2,986,618 (52.73 %)	3,148,409 (48.76 %)

		PE1	PE2	PE3	PF1	PF2	PF3
reads used in **A0**		118,935 (4.08 %)	204,520 (4.04 %)	208,138 (4.02 %)	157,014 (4.36 %)	226,488 (4.40 %)	230,139 (4.38 %)
reads used in **A1**		280,432 (9.61 %)	481,748 (9.53 %)	487,064 (9.40 %)	357,641 (9.92 %)	512,202 (9.95 %)	515,080 (9.80 %)
reads used in **A2**		405,574 (13.90 %)	698,160 (13.81 %)	708,636 (13.68 %)	507,637 (14.08 %)	725,237 (14.09 %)	730,624 (13.89 %)
mapped reads to *A. thaliana* ESTs (**AL0**)	mapping with no mismatch against 33,602 cDNA sequences of TAIR10 database	483,239 (15.22 %)	825,466 (14.97 %)	834,303 (13.30 %)	549,849 (14.50 %)	785,646 (13.87 %)	798,432 (12.37 %)
mapped reads to *A. thaliana* ESTs (**AL1**)	mapping with one mismatch against 33,602 cDNA sequences of TAIR10 database	1,094,779 (34.49 %)	1,875,483 (34.01 %)	1,891,310 (30.16 %)	1,270,380 (33.50 %)	1808647 (31.94 %)	1,830,863 (28.36 %)
mapped reads to *A. thaliana* ESTs (**AL2**)	mapping with two mismatches against 33,602 cDNA sequences of TAIR10 database	2,047,371 (64.50 %)	3,537,753 (64.16 %)	3,619,501 (57.71 %)	2,432,940 (64.16 %)	3,473,952 (61.34 %)	3,548,393 (54.96 %)

	PE1	PE2	PE3	PF1	PF2	PF3
reads used in **AL0**	352,634 (11.11 %)	605,585 (10.98 %)	614,769 (9.80 %)	420,759 (11.10 %)	605,670 (10.69 %)	615,570 (9.53 %)
reads used in **AL1**	879,070 (27.69 %)	1,511,490 (27.41 %)	1,531,637 (24.42 %)	1,053,246 (27.77 %)	1,506,268 (26.60 %)	1,525,907 (23.63 %)
reads used in **AL2**	1,393,590 (43.90 %)	2,407,128 (43.65 %)	2,447,617 (39.03 %)	1,691,090 (44.60 %)	2,415,751 (42.65 %)	2,456,724 (38.05 %)
